# Sleeplessness and Insanity

**Published:** 1881-12

**Authors:** 


SLEEPLESSNESS AND INSANITY.

There are more insane people in the United States than in any other part of the world, by fifty per cent. "I can't sleep" is a complaint becoming ever day more familiar to the city physician, and sleeplessness always precedes the ordinary forms of insanity; on the other hand an improvement in the ability to sleep is a certain indication of coming restoration to reason. The want of ability to sleep well, soundly and connectedly, should always meet with prompt attention, to prevent its becoming a habit, a second nature. The speediest method of doing this is to break up the present associations, whatever may be the sacrifice; get some different employment, something more active and stirring. The next best thing is a long journey on horseback, with a good companion; a journey which has an end in view, the selection or location of lands for investment, camping out for months together as far as is practicable from human habitations, relying for provisions wholly on what can be caught or hunted. Visiting new and strange countries is another method of breaking up the treadmill sameness of some kinds of business. The great point for those who cannot sleep satisfactorily is to be a large portion of the time in the open air, and to be occupied in a way to bring into activity other muscles and other mental operations; and in proportion as they are of pleasurable and absorbing interest, so much the happier will be the effect, and the more speedy will be the return to that "balmy sleep," the very thought of which, as enjoyed in youth, is a happiness. Neither money nor medicine can procure healthful sleep; it can only be had in all its deliciousness by large out-door activities or homely toil.

---

We have copied the above article from Dr. Hall's Health at Home, the most complete work on medicine for the family that has ever been published. Price, $4. We send this book, post-paid, to any address on receipt of the price, or we will send the book and Hall's Journal of Health one year for $5.

---

Said the lecturer, "The roads up these mountains are too steep and rocky for even a donkey to climb; therefore I did not attempt the ascent."

"Your meal is ready," said the waiter to Hayseed, just from the rural districts. "Meal!" exclaimed Hayseed, contemptuously. "Do yer think I'm a hoss? Get me some corned beef and cabbage, young man."

